# Detection of gastrointestinal parasites in small-scale poultry layer farms in Leyte, Philippines

**DOI:** 10.14202/vetworld.2018.1587-1591

**Published:** 2018-11-19

**Authors:** Rochelle Haidee D. Ybañez, Kurt Jimwell G. Resuelo, Ara Patrice M. Kintanar, Adrian P. Ybañez

**Affiliations:** 1 Department of Biology and Environmental Science, College of Science, University of the Philippines Cebu, Gorordo Avenue, Lahug, Cebu City, Philippines; 2 Unit for Host Defense, National Research Center for Protozoan Diseases, Obihiro University of Agriculture and Veterinary Medicine, Obihiro City, Hokkaido, Japan; 3 Department of Clinical Veterinary Science, College of Veterinary Medicine at Barili Campus, Cebu Technological University, Barili, Cebu, Philippines

**Keywords:** gastrointestinal parasites, layer chickens, Leyte, Philippines, small-scale farms

## Abstract

**Background:**

Gastrointestinal (GIT) parasites can affect poultry productivity by compromising its health. It is well studied in other countries, but the documented reports in the Philippines have been limited.

**Aim:**

The aim of the present study was to evaluate the presence of GIT parasites in selected small-scale poultry layer farms in Leyte, Philippines.

**Materials and Methods:**

A total of 243 stool samples from eight small-scale poultry layer farms in Leyte, Philippines, were examined for GIT parasites using floatation and sedimentation technique. Profile parameters were also obtained. Fecal samples were collected and analyzed using floatation and sedimentation techniques. Statistical significance between GIT parasite positivity and profile parameters was determined using Chi-square test.

**Results:**

GIT parasites were detected in 92.2% of the samples (24.7% with single infection, 42.0% with 2-3 parasites, and 25.5% with three or more parasites). The common parasites detected were *Ascaridia* spp. (41.2%), *Heterakis* spp. (59.3%), *Capillaria* spp. (10.7%), *Eimeria* spp. (43.2%), and *Strongyloides* spp. (74.1%). Some profile parameters, including farm location, years in business, number of workers, nearby water system, the practice of fecal cleaning, and presence of other animals, were found to be significantly associated with GIT positivity.

**Conclusion:**

GIT parasites were detected in the poultry of small-scale layer farms in selected areas in Leyte, Philippines. This finding calls for the importance of routine GIT parasite monitoring and the implied need for regular deworming or dewormer rotation in the area.

## Introduction

Poultry layer production is presently among the fastest growing industries in the Philippines. Chicken meat and eggs have also become one of the most popular food commodities being vital sources of protein in the human diet [1]. Compared to other livestock, the production cost of poultry raising per unit is low, and the return on investment is faster and higher [2]. At present, most chickens and eggs sold in the market are produced by local, small-scale poultry layer farms [3].

Despite the growth of the Philippine poultry industry, the economics of poultry farming is still hampered by frequent outbreaks of diseases. Of these diseases, parasitic infections by gastrointestinal (GIT) parasites cause more considerable damage and massive economic losses due to malnutrition, decreased feed conversion, weight loss, lowered egg production, and death in juvenile birds [4]. The common GIT parasites that infect poultry include helminths, cestodes, nematodes, and protozoans, with mixed infections being widespread [5]. These GIT parasites have morphological and physiological features adapted to live longer in their hosts [6]. Risk factors that contribute to the spread of these parasites into the layer chickens include poultry transports, neighborhood infection, unhygienic practices of farms, and the introduction of infected foreign birds [7].

There has been a significant decrease in the prevalence of most of the parasitic diseases in commercial poultry production systems through refined housing, hygiene, and administration [8]. However, parasitic infections are still significant problems in egg-producing small-scale layer farms. The Philippine Statistics Authority [9] reported an increase in the inventory of layer and native chicken in the Philippines compared to the previous year’s inventory. This increase has also increased egg production. The growth of the Philippine poultry industry denotes a continuing need to improve the prevention of chicken parasitic infections for a continuous distribution of quality and safe products. Furthermore, there is a constant need to assess the status of the layer chicken production and the risk factors affecting it.

To the best of the authors’ knowledge, there are no published reports yet regarding the presence of GIT parasites in layer chickens in Leyte, Philippines. The information on the presence of these parasites will be integral in understanding their epidemiology and the necessary preventive measures that can be done. Hence, this study aimed to detect GIT parasites in small-scale poultry layer farms in selected areas in Leyte, Philippines.

## Materials and Methods

### Ethical approval

The procedures performed in this study were guided by the principles of animal welfare, Animal Welfare Act of the Philippines (RA 8485) and Administrative Order No. 45 of the Bureau of the Animal Industry of the Philippines.

### Informed consent

The farm owners were interviewed but were limited to farm data and not about the farm owners. Only letters of request were sent. In the Philippine laws, informed consent to people are required by ethics if the interview covers that of the personal information of the respondents.

### Selection and profiling of small-scale layer farms and animals

This descriptive, analytical study was conducted in eight small-scale layer farms in Leyte, Philippines, which were purposively selected. The study was conducted from April to June 2016. In the Philippines, there is not much difference in the season in the weather conditions. Hence, the season is not seen as a factor. Routine preventive deworming is performed based on age of the bird. Before the actual sampling, letters were sent to each farm requesting approval to conduct the study. The farm owners or workers on duty were then interviewed to obtain the farm and animal profiles. The profile parameters include farm location, farming type and housing elements, deworming program, and management system.

### Fecal sample collection

In as much as we would want to get samples from each bird, the birds are not caged individually in the layer houses, and thus, it may be difficult to ascertain which bird defecated. Thus, stool samples were preferred. Furthermore, in actual practice here in the Philippines, we only do random sampling to diagnose a farm and treat the entire layer house or farm. We do not treat individual animals, especially in worm infestation. Fecal samples were collected from freshly excreted feces or on-ground feces. Approximately 10 g of feces were obtained per sample from different pens (regardless of the number of animals per pen) and were transferred into properly labeled containers. Labeled containers have information on sample number, production phase, date of collection, pen number, and number of heads (per pen). Immediately after collection, 10% formaldehyde was added to the samples and was stored at 4°C until analysis.

### Fecal analysis

Approximately 1 g of each of the preserved fecal samples was processed using sugar solution and distilled water for floatation [10] and sedimentation methods [11], respectively. Examination of parasite eggs was done under a light microscope. Structural and morphometric criteria were utilized during the identification of the eggs [12]. The analysis was limited to the use of fecal samples, and necropsy was not performed to recover worms.

### Data collection, processing, and analyses

Obtained profile and fecalysis results were manually encoded in Microsoft Excel. Descriptive statistics were employed. Statistical significance was determined using Chi-square test. The positivity rate (PR) was computed using the following formula:


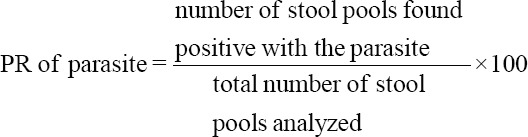


## Results and Discussion

### Profile of selected small-scale layer farms in Leyte

The layer population in the farms ranged from 500 to 4000 heads. Most adapted 3-4 birds per cage and all had slatted floors. Only one farm had free-roaming chickens in the area. Most had two buildings, which had been operating for 2-10 years. The farms had 1-6 workers and were mostly using pellet type of feeds. The majority had no nearby water system with no closed perimeters. Cleaning of feces was usually done every 3-7 days (Table-1). These profile parameters were reported to have a significant association with productivity and GIT parasite presence [3,13-15].

**Table-1:** Profile of selected small-scale poultry layer farms tested for GIT parasites in selected areas in Leyte, Philippines.

Farm	Population/farm	Location	3-4 chickens per cage	Type of flooring	Free-roaming chickens	Number of samples	Number of buildings	Years in operation	Number of workers	Type of feeds	Water system nearby	Farm perimeter type	Fecal cleaning practice
A	500	Baybay	Yes	Slatted	Absent	14	1	4	1	Pellet	No	Closed	1-day interval
B	4000	Baybay	Yes	Slatted	Absent	26	1	10	3	Pellet	No	Closed	3-day interval
C	700	Hilongos	Yes	Slatted	Absent	16	2	4	1	Pellet	No	Open	3-day interval
D	4000	Baybay	Yes	Slatted	Absent	30	2	2	6	Mashed	Yes	Open	Pond type
E	3000	Hilongos	No	Slatted	Present	33	2	4	3	Pellet	No	Open	4-day interval
F	3000	Baybay	Yes	Slatted	Absent	44	2	4	2	Pellet	Yes	Open	7-day interval
G	2500	Baybay	Yes	Slatted	Absent	45	2	5	2	Pellet	No	Open	5-day interval
H	1000	Albuera	Yes	Slatted	Absent	35	1	2	3	Pellet	Yes	Open	7-day interval

GIT=Gastrointestinal

It was noted during fecal collection that all farms reportedly dewormed their flock every 3-6 months. Parasites may acquire resistance to therapeutic drugs made to combat their spread in layer chickens, thereby increasing the incidence of infection [15]. Despite regular deworming, GIT parasites were still detected in all eight farms.

### GIT parasites detected in layer chickens in Leyte

**Figure-1:**
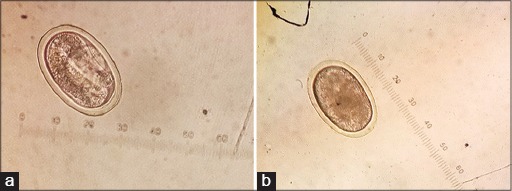
*Strongyloides* (a) and *Ascaridia* (b) spp. eggs detected in layer chickens in Leyte, Philippines.

A total of 243 samples were collected from the eight participating poultry layer farms in Leyte. Fecalysis results revealed that GIT parasites were detected in 92.2% of the samples (24.7% with single infection, 42.0% with 2-3 parasites, and 25.5% with three or more parasites). The GIT parasites detected were *Ascaridia* spp. (41.2%), *Heterakis* spp. (59.3%), *Capillaria* spp. (10.7%), *Eimeria* spp. (43.2%), and *Strongyloides* spp. (74.1%) (Table-2 and Figure-1).

**Table-2:** Detection rate (%) of GIT parasites in the selected farms in Leyte, Philippines (n=243 stool pools)

Parasite	Farm								Total (n=243)

	A (n=14)	B (n=26)	C (n=16)	D (n=30)	E (n=33)	F (n=44)	G (n=45)	H (n=35)		
*Ascaridia* spp.	14.3	84.6	81.3	56.7	27.3	56.8	0.0	34.3	41.2
*Heterakis* spp.	57.1	88.5	75.0	70.0	75.8	63.6	33.3	34.3	59.3
*Capillaria* spp.	0.0	7.7	0.0	3.3	12.1	20.5	20.0	2.9	10.7
*Eimeria* spp.	35.7	76.9	75.0	56.7	24.2	61.4	13.3	28.6	43.2
*Strongyloides* spp.	35.7	76.9	62.5	96.7	66.7	95.5	64.4	65.7	74.1

GIT=Gastrointestinal

*Strongyloides* spp. eggs had the highest PR among the parasites detected (Table-2). It is a soil-transmitted, parasitic, and zoonotic nematode that invades the ceca. *Strongyloides* spp. has been one of the highly isolated nematode parasites in chickens [16].

The second most detected, *Heterakis* spp., has been isolated and identified in various prevalence studies on GIT parasites of chickens [17]. Histopathological findings discovered that *Heterakis* infestation in the GIT caused severe damage to the cecal architecture, necrosis of lamina propria, and the overall destruction of intestinal glands. Infected cecum exhibited muscular alterations and tearing [18].

Coccidia, such as *Eimeria* spp., are one of the most important protozoan parasites of poultry, both in terms of distribution, frequency, and economic losses [19]. They are passed through a chicken’s droppings and attach itself to the intestinal lining. Outward signs of coccidiosis include droopiness, loss of appetite, blood or mucus in the feces, diarrhea, dehydration, and even death [20,21]. Many farms are vulnerable to the transmission of this parasite through the transportation of personnel and equipment. New farms alike have a high probability of acquiring this parasite within a few weeks after poultry introduction [19].

*Ascaridia* spp., on the other hand, are commonly reported parasitic and zoonotic nematodes of the chicken that lives in the small intestine. Several studies have found it to be the most prevalent and pathogenic nematode species of the chicken [22]. The high frequency of this parasite is likely due to its direct life cycle. Ingestion of water and food contaminated by infective eggs leads to the development of the egg into its larval stage when reaching the small intestine [23]. Parasite infestation is characterized by gross lesions in the small intestine, hemorrhage, intestinal wall thickening, the presence of nodules, and necrotizing enteritis [24].

### Statistical analysis of profile parameters and GIT parasite positivity

Selected profile parameters including farm location, years in business, number of workers, proximity to a water system, frequency of fecal cleaning, and presence of other animals were found significantly associated (p<0.05) with GIT parasite positivity (Table-3). Other farm profiles including age, flooring, caging density and presence of free-roaming chickens, type of feeds, farm system, the presence of insects, and dead/sick chickens showed no significant association to the frequency of detected parasites.

**Table-3:** Statistical analysis results between profile and GIT parasite positivity.

Parameter	p-value
Farm location	0.000**
Flooring	1.000
Caging	0.747
Free roaming	0.747
Number of buildings	0.334
Years in business	0.021*
Number of workers	0.042*
Type of feeds	0.086
Water system nearby	0.012*
Farm system	0.376
Frequency of fecal cleaning	0.001**
Presence of other animals	0.007**
Presence of insects	0.376
Presence of dead/sick chickens	0.334

* Significant,

** Highly significant, GIT=Gastrointestinal

The farm location influencing the presence of parasites reveals that there might be different management practices or parasite biodiversity in the area. However, farm location may not be a significant factor if the characteristics of the farms are similar [25]. On the other hand, the number of years the farm is in business was found to be another significant factor. The birds in the farms may be older since the prevalence of parasitic infections can accumulate with increasing age of generations [26]. Furthermore, more years in business could entail more generations of birds that share and accumulate similar parasitic loads.

The number of workers on the farm may also affect GIT positivity. The more workers in the farm, the more people are there to take care of the cleanliness of the farm and care for the birds and the basic needs [27]. Similarly, maintenance of flock density, chicken segregation, and control of environmental factors, such as water and food, are all accomplished through the workers [28].

The proximity to a water source, as well as the presence of other types of animals, such as pigs and dogs, may also influence the existence of parasites [29]. Other animals on the farm can be sources of contamination, especially when problems of farm management and poultry handing are rampant [30].

## Conclusion

GIT parasites were detected in all farms at a high level (92.2%). The most common parasites detected were *Ascaridia* spp. (41.2%), *Heterakis* spp. (59.3%), *Capillaria* spp. (10.7%), *Eimeria* spp. (43.2%), and *Strongyloides* spp. (74.1%). Some profile parameters, including farm location, years in business, number of workers, nearby water system, the practice of fecal cleaning, and presence of other animals, were found to be significantly associated with GIT positivity. This study is the first survey on GIT parasites in small-scale poultry layer farms in selected areas in Leyte, Philippines.

## Authors’ Contributions

RHDY, KJGR, and APMK contributed equally to the study. APY conceptualized the study and analyzed and corrected the manuscript. All authors have read and approved the final manuscript.
